# The role, regulatory mechanisms, and therapeutic implications of FTO in gastrointestinal cancer

**DOI:** 10.1016/j.gendis.2026.102122

**Published:** 2026-03-06

**Authors:** Xinxin Zhang, Yufeng Han, Huimin Yin, Zhenjian Zhuo, Jing He

**Affiliations:** a Department of Pediatric Surgery, Guangzhou Institute of Pediatrics, Guangdong Provincial Key Laboratory of Research in Structural Birth Defect Disease, Guangzhou Women and Children's Medical Center, Guangzhou Medical University, Guangzhou, Guangdong 510623, China; b Brain Function and Disease Laboratory, Shantou University Medical College, Shantou, Guangdong 515041, China

**Keywords:** FTO, Gastrointestinal cancer, Inhibitors, m6A, Posttranslational modifications, Upstream regulators

## Abstract

N6-methyladenosine (m6A) is the most common post-transcriptional modification in mRNA, playing a crucial role in cancer development by modulating RNA stability, translation, and nuclear export. As the first discovered m6A demethylase, FTO catalyzes m6A demethylation in a Fe^2+^/α-KG-dependent manner, functioning as either an oncogene or a tumor suppressor to mediate cancer progression via reducing m6A levels and thereby modulating RNA metabolism. Numerous studies have elucidated the mechanisms by which upstream regulators affect FTO expression, as well as the post-translational modifications that impact its protein stability, translocation, and degradation. Moreover, inhibitors targeting FTO demonstrate significant therapeutic potential. In this review, we summarize recent research on the role and regulatory manner of FTO in gastrointestinal cancer and discuss its potential clinical application in cancer therapy.

## Introduction

In eukaryotes, RNA metabolism relies not only on transcriptional control of genes but also on RNA modifications at the post-transcriptional level. Among the more than 170 known RNA modifications, RNA methylation is the most prevalent, accounting for nearly two-thirds of the total, including N6-methyladenosine (m6A), N1-methyladenosine (m1A), 5-methylcytidine (m5C), and 7-methylguanosine (m7G). To date, m6A is the most extensively studied modification occurring in mRNA, tRNA, lncRNA, microRNA, and circRNA.^1^, ^2^, ^3^, ^4^, ^5^ In 2012, the development of the whole transcriptome m6A detecting method (m6A-seq) provided key tools for the identifying m6A modifications, which accelerated the exploration of the role of m6A in cancers.^6^, ^7^, ^8^ Previous studies have revealed that m6A mainly occurs at the RRm6ACH (R = G or A; H = C, A, or U) motif, and can be recognized by m6A reader proteins to affect RNA fate.^9^, ^10^, ^11^, ^12^, ^13^ Extensive evidence demonstrates that m6A mediates nearly all aspects of RNA metabolism, including mRNA stability, export, translation, splicing, and degradation, which mediates the progression of various cancers.^14^, ^15^, ^16^

Mechanistically, m6A is dynamically regulated by “writers” and “erasers” and recognized by “readers” to mediate RNA fate. The reported m6A methyltransferase complex consists of methyltransferase-like 3/14 (METTL3/14) and WT1-associated protein (WTAP). Among them, METTL3 acts as the catalytic subunit, which deposits m6A modification to the substrate in an S-adenosyl-l-methionine (SAM)-dependent manner.^17^ Structural analysis revealed that METTL3 and METTL14 form a heterodimer modulating the m6A deposition on nuclear RNA.^18^ Previous studies have demonstrated that WTAP plays a role in mediating mRNA splicing and regulating alternative splicing.^19^^,^^20^ Pinget al. revealed that WTAP is essential for the localization of METTL3 and METTL14 into nuclear speckles.^21^ Additionally, METTL16, vir-like m6A methyltransferase-associated (VIRMA), Cbl proto-oncogene-like 1 (HAKAI, zinc finger CCCH-type containing 13 (ZC3H13), and RNA-binding motif protein 15/15B (RBM15/15B) also interact with methyltransferase complex to mediate m6A methylation on specific targets.^22^, ^23^, ^24^, ^25^, ^26^ At present, two AlkB family proteins have been identified as m6A demethylases, including FTO and AlkB homolog 5 (ALKBH5). ALKBH5 primarily recognizes m6A-modified mRNA, while FTO can recognize various types of RNA, including mRNA, snRNA, and tRNA. Moreover, FTO shuttles between the cytoplasm and the nucleus, whereas ALKBH5 is predominantly localized in the nucleus. Additionally, they remove m6A methylation in different ways. FTO first catalyzes m6A to generate N6-hydroxymethyladenosine (hm6A) and N6-formyladenosine (f6A). Subsequently, these two intermediate products are converted to adenosine (A) under conditions at 37 °C.^27^ In contrast, ALKBH5 accomplishes the progression of m6A demethylation in a single step.^28^ Ueda et al found that ALKBH3 specifically recognizes m6A in tRNA within cancer cells.^29^ In addition, the m6A “reader” proteins are crucial regulators that direct m6A-modified RNA into various metabolic pathways. The YTH domain family contains the largest group of m6A readers (YTHDF1/2/3, and YTHDC1/2), which modulate m6A RNA stability, translation, and nuclear export.^11^, ^12^, ^13^^,^^30^

IGF2 mRNA binding protein (IGF2BP1/2/3) recognizes m6A-modified RNA to enhance mRNA stability and translation.^31^ The heterokaryotic nuclear RNA protein family (HNRNPC, HNRNPG), acting as readers of m6A RNA, mediates pre-RNA alternative splicing and microRNA processing.^32^, ^33^, ^34^

In the course of m6A-related studies, the identification of the first m6A demethylation enzyme, alpha-ketoglutarate-dependent dioxygenase (FTO), represents a significant landmark discovery. This finding confirms the dynamic nature of m6A and positions it as one of the most prominent targets in epigenetics.^35^ Meanwhile, FTO is one of the most extensively studied m6A regulators in cancers. Considering its widespread distribution, substrate diversity, and functional variability, this review summarizes recent advances in understanding the role and molecular mechanisms of FTO in gastrointestinal cancer (GICs), including the known upstream regulators and posttranslational modifications of FTO, and discusses the progress in the development of small-molecule inhibitors targeting FTO for cancer treatment.

## Structure and function of FTO

**Figure 1:**
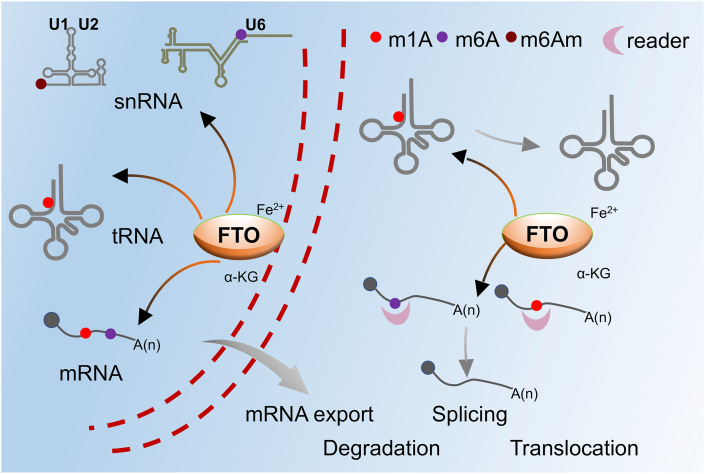
The substrate and function of FTO in RNA metabolism. FTO recognizes mRNA, tRNA, and snRNA, and catalyzes the demethylation of m1A, m6A, and m6Am in a Fe^2+^/α-KG-dependent manner, which mediate RNA metabolism, including mRNA export, splicing, degradation, and translocation.

FTO, a member of the AlkB family, catalyzes the demethylation of substrates in a Fe^2+^/α-ketoglutarate (α-KG)-dependent manner. The reported catalytic substrates of FTO include m6A, m1A, and the N6, 2′-O-dimethyladenosine cap (m6Am) modification. In the nucleus, FTO demethylates m6A and m6Am in small nucleus RNA (snRNA), N1-methyladenine (m1A) from tRNA, and m6A in mRNA. When localized in the cytoplasm, FTO can demethylate m6A and m6Am in mRNA and m1A from tRNA 36, 37, 38 (Fig. 1). Crystal structure analysis has revealed that FTO consists of an Alkb-like domain (NTD, residues 32–326) and a carboxy-terminal domain (CTD, resides 327–498). The NTD serves as the catalytic core of FTO, containing the binding sites for Fe^2+^ and N-oxalylglycine. The CTD stabilizes the conformation of the NTD, thereby maintaining FTO demethylase activity.^39^ The R316Q mutation has been identified a key site for enzymatic activity through genetic analysis of a family with growth retardation and polymalformative syndrome.^40^

## The role of FTO in gastrointestinal cancer

As one of the most widely reported m6A medication regulators, FTO has been verified to be tightly involved in the development of various cancers by mediating RNA stability, nuclear export, and translation. GICs represent one of the foremost contributors to cancer-related deaths across the globe.^41^ Here, we summarize recent studies elucidating the role of FTO as both a pro-tumor and tumor suppressor factor in GICs (Table 1).

**Table 1:** Role of FTO in gastrointestinal cancer.

Cancer	Role	Upstream	Target	Reader	Function
GC	Oncogene		PI3K /Akt		GC malignancy^42^
GC	Oncogene		MOXD1		GC malignancy^43^
GC	Oncogene		Caveolin-1		GC tumor growth^44^
GC	Oncogene		MAP4K4		Tumorigenesis and metastasis^45^
GC	Oncogene		circFAM192A		GC cells proliferation^46^
GC	Oncogene		SP1		Invasion and LNM^47^
GC	Oncogene	HDAC3 /FOXA2	MYC		Tumorigenesis^48^
GC	Oncogene		DDIT3		Chemosensitivity^49^
GC	Suppressor	MYC	FOS	IGF2BP1/2	Migration and invasion^50^
LC	Oncogene		GPNMB	YTHDF2	Proliferation, migration and invasion^51^
LC	Oncogene	FTO-IT1	PKM2/GLUT1	YTHDF2	Glycolysis and proliferation^52^
LC	Oncogene		ULK1	YTHDC2	Autophagy^53^
LC	Oncogene		AMD1	SOX2	Stem-cell property^54^
LC	Suppressor		ERBB3/TUBB4A		Proliferation, migration, and tumorigenesis^55^
LC	Suppressor	CircGPR137B			Cell growth, tumorgenetics^56^
LC	Suppressor		CUL4A		Glucose metabolism^57^
CRC	Oncogene	AKT	GPX4	YTHDF2	Protect from ferroptotic cell death^58^
CRC	Oncogene		PD-L1		Impact PD-L1 expression^59^
CRC	Oncogene		ZNF687		Tumor growth, metastasis, and angiogenesis^60^
CRC	Oncogene		G6PD/PARP1		Chemo-Resistance^61^
CRC	Oncogene		SIVA1		Chemo-Resistance^62^
CRC	Suppressor		HK2	IGF2BP2	Glycolysis^63^
CRC	Suppressor	microRNA-96	MYC		Proliferation and apoptotic^64^
PC	Oncogene		PDGFC	YTHDF2	Proliferation^65^
PC	Oncogene		LINC01134		Chemoresistance^66^
PC	Oncogene		miR-383–5p	IGF2BP1	Cell viability, metastasis, stemness^67^
PC	Oncogene	USP7	NEDD4		Chemoresistance^68^
PC	Oncogene		TFPI-2	YTHDF1	Growth, migration, and invasion^69^
PC	Oncogene	GATA6-AS1	SNAI1		Epithelial-mesenchymal^70^
PC	Suppressor		PJA2	YTHDF2	Proliferation, invasion and metastasis^71^

GC, gastric cancer; CRC, colorectal cancer; LC, liver cancer; PC, pancreatic cancer.

## Gastric cancer

Gastric cancer (GC) ranks as the fifth most prevalent and fatal cancer globally. In 2022, over 968,000 new cases of GC were diagnosed, resulting in nearly 660,000 deaths.^72^ Nonetheless, the exact mechanisms contributing to the high prevalence of GC remain poorly understood. Therefore, identifying key molecules that influence the progression of GC is critical for the development of potential therapeutic approaches. Bioinformatics and experimental studies confirmed a significantly correlation between the FTO gene and gastric cancer at both the genetic and transcriptional levels.^73^^,^^74^ GC patients with up-regulated FTO present markedly lower overall survival rates, which are mediated through multiple mechanisms. For instance, Zhu et al and Lai et al found that FTO promotes GC malignancy by upregulating the PI3K/Akt signaling and DBH-like monooxygenase 1 (MOXD1) expression, respectively.^42^^,^^43^ Yin et al found that FTO promotes GC progression by suppressing MAP4KA degradation and activating MAPK/JNK signaling pathways.^45^ As a prognostic marker in GC, FTO reduces caveolin-1 mRNA by enhancing its degradation in an m6A-dependent manner, which promotes GC cell malignancy and tumor growth by inducing mitochondrial fusion and metabolism.^44^ Besides the most common mRNA, FTO could modulate circRNA fate to affect GC progression. Zhou, et al proved that FTO could recognize circFAM192A and remove its m6A methylation, thereby protecting it from degradation and enhancing the stability of interacting protein leucine transporter solute carrier family 7 member 5 (SLC7A5). As a result, the amount of SLC7A5 significantly increased on the membrane, activating the mTOR signaling pathway to promote GC cell proliferation.^46^ Functionally, FTO has been demonstrated to mediate proliferation and migration of various types of GC cells^75^^,^^76^ and to enhance cellular malignancy by promoting the stemness of GC cells.^77^ Recently, Zeng et al reported that FTO correlates with tumor size, advanced nerve invasion, and lymph node metastasis (LNM) in GC patients. They revealed that FTO increases the levels of specificity protein 1 (SP1) and Aurora Kniase B by removing its methylation, leading to the dephosphorylation of P53 and promoting GC tumorigenesis and progression.^47^

Viral infection is a significant factor in the onset of gastric cancer,^78^ and FTO regulates the efficiency of viral infection, thereby modulating the development of gastric cancer. Gui et al found that *Helicobacter pylori* infection significantly increases both the mRNA and protein levels of FTO in both human gastric mucosal epithelial cells and Mongolinan gerbil gastric tissues. The knockdown of FTO significantly reduces the abilities of migration and invasion in *Helicobacter pylori* -infected GC cells, suggesting that FTO may serve as a potential therapeutic target to reduce virus-infected GC malignancy.^79^ Interestingly, FTO is also highly expressed in Epstein–Barr virus-associated gastric cancer (EBVaGC), but it restrains EBVaGC cell migration, invasion, and tumor metastasis. FOS was identified as a critical downstream target of FTO by MeRIP-seq, while IGF2BP1/2 binds to the m6A modification of FOS nascent transcripts to maintain its mRNA stability. Its upstream regulator EBV activates myelocytomatosis viral oncogenehomolog (MYC) binding to the promoter of FTO to elevate FTO expression in EBVaGC cells.^50^

In addition, several upstream regulators, including transcription factors and clinical drugs, can regulate the development and treatment efficacy of gastric cancer by targeting FTO. Yang et al found that the transcription factor A2 (FOXA2) binds to the FTO promoter, leading to a decrease in FTO expression in GC. Mechanistically, FOXA2 inhibits FTO to remove the m6A methylation of MYC), thereby stabilizing MYC mRNA and promoting gastric cancer progression. Furthermore, interactor protein of FOXA2, HDAC3, also regulates GC development within the FTO/m6A/MYC signaling pathway through degrading FOXA2.^48^ The clinical drug omeprazole has been widely utilized in the treatment of gastropathy, including gastritis and gastric ulcers. Feng et al found that omeprazole enhances chemosensitivity via reducing FTO levels in GC cells. The inhibition of FTO improves the mROTC1 signaling pathway activity through mediating apoptosis-related tumor suppressor gene DDIT3 mRNA levels in an m6A-dependent manner.^49^ The aforementioned studies demonstrate a strong connection between FTO and GC, indicating that FTO may be a potential target for prognostic assessment and therapeutic intervention in GC.

## Liver cancer

**Figure 2:**
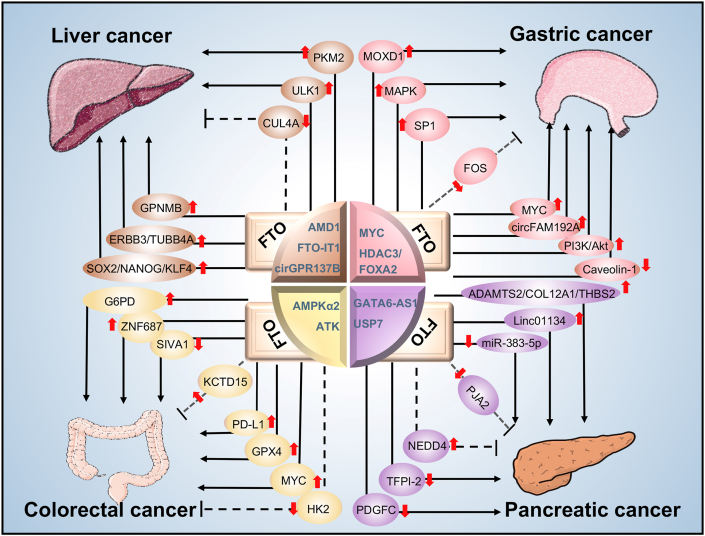
The targets and roles of FTO in gastrointestinal cancer. In liver cancer, FTO promotes cancer progression via downregulating ERBB3/TUBB4A and upregulating GPNMB, ULK1, PKM2 and SOX2/NANOG/KLF4, while it suppresses tumor progression via downregulating CUL4A. FTO-IT1, AMD1 and cirGPR137B target FTO and modulate its expression. In gastric cancer, FTO promotes cancer development by downregulating the levels of caveolin-1 and upregulating MOXD1, MAPK, SP1, MYC, PI3K/Akt and circFAM192A, while suppresses tumor development via decreasing FOS. Upstream regulators like MYC and HDAC3/FOXA2 modulate FTO expression. In colorectal cancer, FTO promotes cancer development by downregulating SIVA1 and upregulating G6PD, ZNF687, PD-L1, GPX4 and MYC, while suppresses cancer development by downregulating HK2 and upregulating KCTD15. AMPKα2 and AKT as upstream regulators affect FTO expression. In pancreatic cancer, FTO promotes cancer development via upregulating ADAMTS2/COL12A1/THBS2, Linc01134 and miR-383–5p, and downregulating TFPI-2 and PDGFC, while suppresses cancer development via upregulating PJA2 and NEDD4. Upstream regulators, USP7 and GATA6-AS1, control FTO expression.

Liver cancer is a highly malignant tumor and ranks as the third leading cause of cancer-related mortality worldwide.^72^ Hepatocellular carcinoma (HCC) is the most prevalent type of liver cancer, accounting for nearly 90% of cases. Although significant progress has been made in liver cancer research over the years, the molecular mechanisms that drive tumorigenesis and development remain unclear. In recent years, numerous studies have confirmed the significance of FTO in liver cancer (Fig. 2).^80^^,^^81^ On one hand, FTO plays a promoting-tumor role in liver cancer in various hepatic cell types. PKM2 was identified as a functionally downstream target of FTO in HCC using m6A-seq and RNA-seq analyses, confirming that FTO activates the m6A demethylation of PKM2 and accelerates its translation, thereby promoting HCC malignancy *in vitro*.^82^ Besides liver cancer cells, FTO is also upregulated in bile duct ligation (BDL)-induced hepatic fibrosis mice. Mechanistically, FTO demethylates m6A modification of ULK1, enhancing autophagy and triggering the activation of hepatic stellate cells, which leads to live fibrosis in a YTHDC2-dependent pathway.^53^ The role of FTO in HCC has been further validated in HepG2 cells. A study by Ye et al demonstrated that silencing FTO suppresses cell proliferation and colony formation in HepG2 liver cancer cells. Clinically, low expression levels of FTO in patients is indicative of a favorable prognosis.^83^ On the other hand, compared to the adjacent non-tumor tissue, FTO levels are significantly decreased in HCC tissues, and patients with lower FTO expression exhibit shorter overall survival.^84^ Erb-b2 receptor tyrosine kinase 3 (ERBB3) and human tubulin beta class Iva (TUBB4A) were identified as the targets of FTO by integrating MeRIP-seq with RNA-seq. Inhibition of FTO with the inhibitor FB-23 leads to a significant decrease in the mRNA levels of ERBB3 and TUBB4A, resulting in the suppression of Akt-mTOR signaling and impairing proliferation, migration, and tumorigenesis of HCC.^55^ An opposite viewpoint suggests that FTO plays a protective role in liver carcinogenesis, as evidenced by studies indicating that FTO-knockout abrogates the liver damage during the tumor-initiation phase in mice subjected to long-term diethylnitrosamine (DEN) treatment. Additionally, CUL4A has been identified as a functional target of FTO, with the knockdown of CUL4A reversing the hepatocyte proliferation induced by the loss of FTO.^57^

To better understand the regulatory network of FTO in liver cancer, several studies have explored its upstream regulators and interaction proteins. Bian et al found that S-adenosylmethionine decarboxylase proenzyme (AMD1) enhances the interaction of (Ras GTPase-activating-like protein 1) IQGAP1 with FTO, resulting in decreased ubiquitination and increased FTO levels. The up-regulated FTO encourages the expression of SRY-box transcription factor 2 (SOX2), Kruppel like factor 4 (KLF4), and NANOG, thereby enhancing the expression of pluripotency factor and the stem cell-like properties of HCC.^54^ Consistent with the previous study, Wang et al confirmed that FTO facilitates HCC progression through mediating PKM2. They also found that the up-regulated FTO is stabilized by lncRNA FTO intronic transcript 1 (FTO-IT1), which recruits interleukin enhancer binding factor 2/3 (ILF2/ILF3) to its 3′ Untranslated region (UTR), leading to the overexpression of glycolysis associated genes (GLUT1/PKM2/c-Myc). Moreover, YTHDF2 mediates the mRNA degradation of these genes to enhance glycolysis in HCC.^52^ Beyond the unidirectional regulators, Liu et al found a circGPR137B/miR-4739/FTO feedback loop that inhibits cell growth in HCC. They revealed that circGPR137B co-localizes with miR-4739 to increase the expression of FTO. In turn, FTO demethylates the m6A modification of circGPR137B, and elevates its expression.^56^ In conclusion, these studies suggested the pivotal role of FTO on liver cancer, and insights into its regulatory mechanisms present potential therapeutic targets.

## Colorectal cancer

Although significant advances have been established in diagnosis and therapy, colorectal cancer (CRC) remains the third most common cancer and second leading cause of cancer mortality worldwide. Although several studies have revealed an association between variants of the FTO gene and CRC, the underlying mechanism is still unclear.^85^, ^86^, ^87^ While the significant role of FTO in colon cancer has been confirmed in multiple studies, a consensus on its implications has yet to be reached (Fig. 2). Tsuruta et al found that FTO is highly expressed in HCT116 cells and regulates programmed cell death-ligand 1 (PD-L1) mRNA through an m6A modification-dependent manner.^59^ Through RIP and MeRIP-seq assay, zinc finger protein 687 (ZNF687) was identified as a direct target of FTO. The overexpression of FTO strengthened the mRNA stability of ZNF687, thereby aggravating tumorigenesis and metastasis via the Wnt/β-catenin pathway in CRC.^60^ Chemical resistance is one of the major impediments for colon cancer therapy. Wang et al found that FTO strengthens glucose-6-phosphate dehydrogenase expression to balance intracellular reactive oxygen species and mediates PARP1 mRNA to maintain the genome instability, which contributes to chemotherapy resistance and promotes CRC progression.^61^ Lin et al found that FTO is up-regulated in human 5-fluorouracil (5-FU)-resistant colon cancer, and FTO depletion significantly decreases cell proliferation, colony formation, and metastasis. SIVA1 was identified as another target of FTO in 5-FU-resistant CRC, whose expression is inversely correlated with FTO and determines the m6A-dependent 5-FU sensitivity in CRC cells.^62^ Beyond the above findings, recent studies have also revealed the anti-tumor role of FTO in colon cancer. Zhang et al found that FTO expression was markedly decreased in CRC tissue compared to para-carcinoma tissues. The expression of KCTD15, a downstream gene of FTO, significantly increased in an m6A-dependent manner, which is recognized with YTHDF2 and promotes p53 degradation to suppress CRC progression.^88^ Subsequently, Ye et al found that FTO and ALKBH5 jointly modulate HK2 mRNA levels in an m6A dependent manner to activate the FOXS signaling pathway. IGF2BP2 recognizes FOXS to enhance the CRC cell proliferation.^63^ In addition, Relier et al found that cytoplasmic FTO reduces the cancer stem cells potential in CRC through mediating N6,2′-O-dimethyladenosine (m6Am) levels, thereby inhibiting tumorigenicity and chemoresistance.^89^

Considering the significance of FTO in CRC under various conditions, emerging studies have focused on the upstream regulators. Yue et al found that microRNA-96 facilitates CRC cells malignant through suppressing AMPKα2 levels. FTO was identified as a downstream target of AMPKα2, which induces cell growth and invasion by upregulating MYC in an m6A-dependent manner.^64^ Recent studies have confirmed that ferroptosis is a key mechanism regulating tumor development.^90^ Zhang et al reported that FTO expression is downregulated with AKT, which inhibits the proliferation of CRC cells and induces ferroptosis. FTO is involved in this process by enhancing the m6A methylation of GPX4, which is recognized by YTHDF2 and promotes the mRNA degradation of GPX4.^91^

## Pancreatic cancer

Pancreatic cancer (PC) is asymptomatic in early stage and quickly invades surrounding organ cancer, leading to a highly fatal prognosis with a five-year survival rate of nearly 10% in the USA.^92^^,^^93^ Due to rising mortality, PC is projected to become the second leading cause of tumor-induced death by 2030.^94^ Therefore, it is necessary to identify critical regulators that modulate PC progression to develop therapies. As one of the most widely studied RNA modifications, m6A has been reported to be involved in regulating PC development, with FTO being crucial in this process^95^^,^^96^ (Fig. 2). Beside, meta-analysis results show that *FTO* gene polymorphism rs9939609 T/A may be a risk factor in pancreatic cancer,^97^ and multiple studies showed the consistent oncogene role of FTO in PC. For example, Wang et al revealed that FTO increases the expression of extracellular matrix (ECM) genes, such as *ADAMTS2*, *COL12A1*, and *THBS2,* to enhance cell migration and invasion.^98^ Tan et al observed that FTO mediates the activation of platelet-derived growth factor C (PDGFC) in an m6A-YTHDF2 dependent manner, thereby promoting PC tumorigenesis.^65^ Moreover, NEDD4 is identified as another downstream target of FTO, which is up-regulated in an m6A-dependent manner to promote gemcitabine resistance through the PI3K/AKT pathway in PC.^68^ Besides mRNA, miRNA progression and lncRNA metabolism were also mediated by FTO to affect PC progression. Zhang et al showed that silencing FTO promotes the processing of miR-383–5p and decreases its expression, which inhibits cell migration, invasion, and stemness in PC.^67^ Lu et al identified Linc01134 as an oncogene that enhances stem cell features and gemcitabine resistance in PC, and FTO maintains Linc01134 mRNA stability through YTHDF2 in m6A-dependent manner.^66^ Consistent with the above trend, Wang et al observed that FTO is upregulated in PC and correlates with poor prognosis. The knockdown of FTO inhibits PC cell proliferation and migration *in vitro*, as well as tumor growth *in vivo*. Mechanistically, FTO triggered m6A demethylation of TFPI-2 to reduce its mRNA stability in an m6A/YTHDF1 manner to facilitate cancer progression.^69^ In addition to the protumor role, the opposing effect of FTO has also reported in PC. Zeng et al explored changes in the mRNA levels of ten key m6A regulators and found downregulation of FTO expression. FTO enhances the mRNA levels of the tumor suppressor *praja ring finger ubiquitin ligase 2* (*PJA2*) in an m6A-YTHDF2-dependent manner, inhibits Wnt signaling, and ultimately suppresses PC growth and metastasis.^71^

Regarding the upstream regulator of FTO in PC, Lin et al found that USP7 binds to FTO, catalyzing its deubiquitination and maintaining its upregulation. Consequently, the increased levels of FTO improve the chemosensitivity of PC to gemcitabine within the PI3K/AKT pathway.^68^ Moreover, Zhou et al showed that GATA6 antisense 1 (GATA6-AS1) acts as an upstream regulator that impairs the oncogenic function of FTO by suppressing its expression. FTO promotes cells growth, invasion, and the epithelial–mesenchymal transition (EMT) process in PC under hypoxia through elevating the mRNA stability of snail family transcriptional repressor 1 (SNAI1).^70^ This mechanism may represent a viable therapeutic target for hypoxic pancreatic cancers.

## FTO's role in the gastrointestinal tumor microenvironment

Modulating the tumor microenvironment represents a significant pathway through which FTO controls the progression of GICs. Recent evidence has revealed that FTO may also regulate the progression of GC via mediating T cell and macrophage activation, as well as the EMT.

Chen et al identified glycoprotein non-metastatic melanoma protein B (GPNMB) as a target of FTO, which binds to the surface receptor SDC4 on CD8^+^ T cells to inhibit their activation in HCC. Targeting the FTO/GPNMB axis has been shown to significantly enhance immune activation while suppressing tumor growth and metastasis.^51^ GU et al found that FTO influences both M1 and M2 macrophages in mouse macrophage cells through suppressing the NF-κB signaling pathway and the expression of STAT1 and STAT6.^99^ In triple-negative breast cancer (TNBC), FTO demethylates the secretory protein ANGPTL4, which promotes M2 macrophage polarization through paracrine signal that drives TNBC development.^100^ In addition, Garg et al revealed that silencing FTO impairs tumorigenic ability and cancer stem cell expression via EMT regulation in PC.^101^ Although FTO does not directly modulate mRNA levels of EMT/ECM in TNBC,^102^ it can catalyze the demethylation of m6A on HBEGF and inhibit its degradation, thereby facilitating EMT in GC cells.^103^

## Upstream regulators of FTO

**Figure 3:**
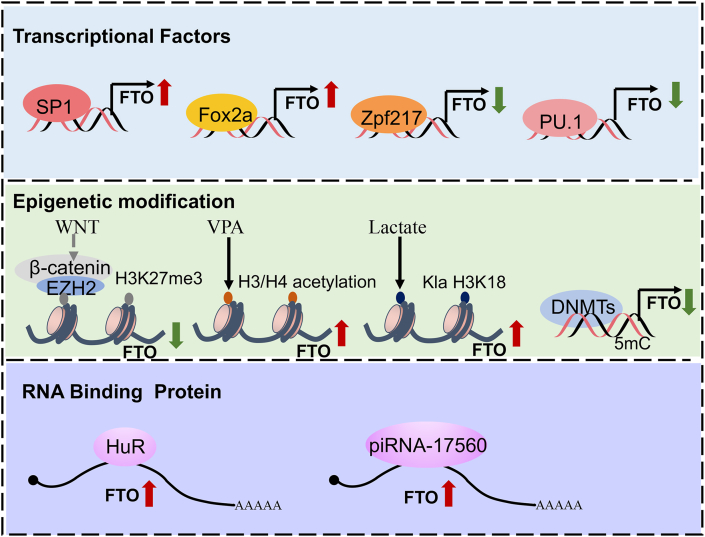
The upstream regulators of FTO. The transcription factors, SP1 and Fox2a, bind to the FTO promoter to enhance its expression, while Zpf217, PU.1 and DNMTs inhibit its expression. HuR and piRNA-17560 recognize and stabilize FTO mRNA. VPC increases H3/H4 acetylation to promote FTO expression. WNT induces β-catenin and EZH2 binds to FTO and decreases its transcription.

Given the crucial role of FTO in cancer development, an increasing number of studies have focused on the upstream regulatory mechanisms governing expression of FTO (Fig. 3). Transcription factors are among the most significant regulators that bind to the promoter and mediate the expression of target genes.^104^ Before the discovery of the demethylation function of FTO, Guo et al found that the transcription factor Foxa2 negatively modulates the transcription and expression of FTO.^105^ In ischemia-reperfusion kidney cells, the upstream transcription factor SP1 binds to the FTO promoter to enhance its mRNA expression.^106^ Moreover, Song et al showed that zinc finger protein Zfq217 activates FTO expression and decreases m6A modification in 3T3-L1 cells, and Zfq217 interacts with YTHDF2 to maintain the interaction between FTO and m6A sites.^107^ A recent study revealed that transcription factor PU.1 inhibits FTO expression in t(8; 21) acute myeloid leukemia, while AML1-ETO suppresses the function of PU.1 by replacing the coactivator c-Jun from PU.1, leading to the up-regulation of FTO and leukemogenesis of t(8; 21) acute myeloid leukemia (AML) cells.^108^

Besides transcription factors, other factors such as RNA-binding proteins and metabolites are involved in the regulation of FTO expression. Yang et al revealed that human antigen R (HuR) binds to the FTO transcript and enhances its mRNA stability.^109^ Ou et al showed that exosomal piRNA-17560 binds to the 3′ UTR of FTO and alleviates its mRNA decay in breast cancer.^110^ Additionally, Yang et al demonstrated that WNT induces the binding of EZH2 to the FTO-interacting protein β-catenin, while EZH2 enhances H3 lysine 27 trimethylation (H3K27me3) and suppresses FTO expression in lung cancer.^111^ Zhang et al revealed that the dynamic of H3/H4 acetylation promotes FTO expression, which could be enhanced by valproate (VPA).^112^ Whole-genome methylation sequencing reveals that the *FTO* gene is methylated with 5-methylcytosine (5 mC) in alcoholic kidneys, and DNA methyltransferases (DNMTs) inhibit its expression.^113^ A recent study found that lactate drove the formation of histone lactylation H3K18la, leading to the up-regulated expression of FTO (Fig. 3).^114^

## The post-translational modification affects FTO

**Figure 4:**
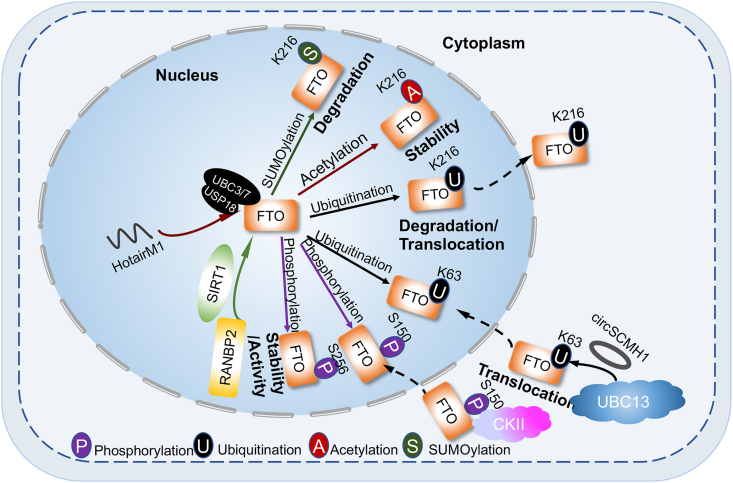
The sites and functions of FTO posttranslational modification. FTO is modified with SUMOylation at K216, acetylation at K216, ubiquitination at K63/K216, and phosphorylation at K256. These modifications modulates its degradation, stability, translocation and activity.

Post-translational modifications (PTMs) provide an elegant and dynamic ways to modulate protein function and enzyme activity.^115^, ^116^, ^117^ Increasing evidence demonstrates that PTMs mediate the function of FTO across various pathways, which may be the potential targets for suppressing cancer development (Fig. 4). A diverse array of PTMs on FTO have been identified, including acetylation, ubiquitination, sumoylation, and phosphorylation. Currently, these modification have been shown to control the degradation, subcellular localization, RNA binding ability, and demethylation activity of FTO (Table 2).

**Table 2:** Regulatory mechanism of PTMs on FTO function.

Modification	Site	Upstream	Enzyme	Function	System	Ref
Acetylation	K88		GCN5	RNA binding ability	Cervical cancer	^118^
	K216	HOTAIRMA		Stability	Nasopharyngeal carcinoma	^119^
Ubiquitination	K216			Localization	Cervical cancer	^120^
	K63	UBC3		Localization	Microvascular ECs	^121^
		USP18		Stability	Neurons	^122^^,^^123^
Sumo2/3	K216		RANBP2	Stability	HCC	^124^
	K216			Stability	BEAS-2B cells	^125^
Phosphorylation	K150		CKII	Stability	HOC313 cells	^126^
			PKCβ	Stability	Hela and 3T3-L1	^127^
	K256			Demethylase activity	Breast cancer	^128^

## Acetylation

Acetylation is a widely-occurring post-translational modification that alters the catalytic activities of enzymes via modulating protein stability, subcellular localization, and interaction.^129^ Our recent study indicated that K88 acetylation of FTO mediates its enzymatic activity and pro-tumor function by enhancing its RNA-binding affinity in HeLa cells.^118^ Mi et al found that the upstream regulator lncRNA HOTAIRMA regulates the acetylation and stability of FTO, resulting in the transition from CD44S to CD44V and improved radioresistance in nasopharyngeal carcinoma. Furthermore, they demonstrated that the acetylation inhibitor A-485 suppresses FTO protein levels in a manner dependent on K216 acetylation in nasopharyngeal carcinoma cells.^119^ The above results indicate that acetylation modifications modulate the binding ability and stability of FTO in cervical cancers, and the protein levels of FTO in nasopharyngeal carcinoma.

## Ubiquitination

Ubiquitination refers to the process by which ubiquitin molecules select and bind to target proteins, playing a crucial role in protein degradation, turnover, and function.^130^ Multiple studies have confirmed the role of ubiquitination in mediating the function of modified m6A regulator proteins.^131^^,^^132^ Zhu et al found that FTO undergoes ubiquitination modification at Lys-216. The K216 mutation inhibits the level of nuclear FTO and blocks its nuclear translocation induced by amino acid starvation treatment in HeLa cells.^120^ In addition, Li et al confirmed the existence of the ubiquitination modification on FTO. Mechanistically, circSCMH1 significantly enhances the interaction between FTO and UBC3, promoting K63 ubiquitination and facilitating the turnover of FTO from the cytoplasm into the nucleus in mouse brain microvascular ECs treated with oxygen-glucose deprivation (OGD).^121^ Recently, it was reported that Ubiquitin-specific peptidase 18 (USP18) enhances FTO protein stability through mediating its de-ubiquitination in PC-12 cells, which activates mitophagy in ischemic stroke and ferroptosis in cerebral ischemia-reperfusion injury.^122^^,^^123^ These findings underscore the critical role of ubiquitination in controlling FTO localization within cervical carcinoma and mouse brain microvascular ECs, as well as its degradation in mouse neurons.

## SUMOylation

As a member of the ubiquitin-like protein family, SUMOylation (small ubiquitin-related modifier) exhibits distinct functions compared to ubiquitination.^133^ Two studies have reported that K216 of FTO is modified with SUMOylation under different conditions. Liu et al found that SUMO2/3 at K216 promotes FTO degradation, leading to the increased m6A levels of the tumor suppressor Guanine nucleotide-binding protein G (o) sub (GNAO1) and reduced mRNA expression, which induces hepatocarcinogenesis. The SUMO2/3 modification is catalyzed by SUMOylation E3 ligase (RANBP2), which is activated by the up-regulation of SIRT1 in HCC.^124^ Zhang et al observed a significant increase in FTO SUMOylation at K216 under arsenic-induced oxidative damage. In line with the above findings, K216 SUMOylation promotes FTO degradation in BEAS-2B cells. Moreover, SUMOylation of FTO regulates the expression of oxidative damage-related genes *HO-1* and *GCLM* in an m6A-IGF2BP3-dependent manner following arsenic treatment.^125^ In summary, the aforementioned studies imply that SUMOylation plays a crucial role in mediating the degradation of FTO protein in HCC and bronchial epithelial cells.

## Phosphorylation

Phosphorylation is one of the most extensively studied PTMs. Abnormal alterations of the phosphorylation pathway lead to a serious outcome in cancer.^134^ Multiple studies have confirmed that phosphorylation can stabilize the key m6A regulator, thereby affecting tumorigenesis.^135^^,^^136^ Casein kinase II (CKII)-catalyzed phosphorylation of FTO at lysine 150 mediates its nucleocytoplasmic shuttling in HOC313 cells.^126^ Tai et al demonstrated that protein kinase Cβ (PKCβ) phosphorylates FTO, thereby inhibiting its ubiquitin-mediated degradation in HeLa and 3T3-L1 cells.^127^ The S256D mutants, which mimics the phosphorylated state of FTO, lead to a significantly reduced m6A demethylation ability of FTO in MDA-MB-231 cells.^128^ These results indicate that phosphorylation modulates FTO levels through blocking its proteasomal degradation in cervical cancer and mouse embryonic fibroblasts cells. Additionally, it affects FTO localization in oral squamous cell carcinoma, and affects its catalytic activity in breast cancer, without altering its localization or protein abundance pathways.

## The development of FTO small molecule inhibitors

**Figure 5:**
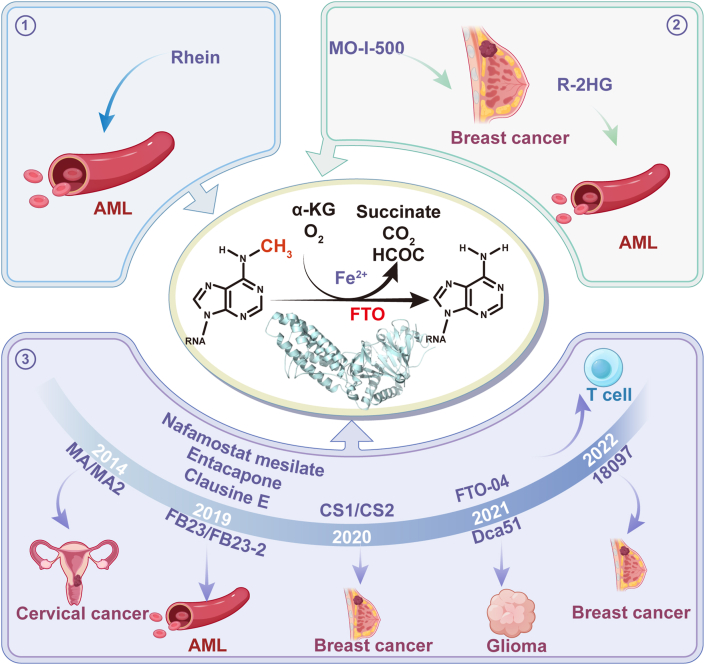
The identified FTO inhibitors for cancer treatment. The design of current FTO inhibitors is based on its demethylation reaction condition or structure characteristics. Rhein is a substrate analog that competitively binds to FTO. MO-I-500 and R-2HG inhibit FTO activity by competing with its essential cofactor, α-ketoglutarate (α-KG). Most inhibitors, including MA/MA2, Entacapone, Clausine E, FB23/FB23-2, CS1/CS2, FTO-04, Dca51 and18097, are designed to directly bind to FTO to improve the specificity.

The abnormal expression of FTO is present in nearly all type of cancers, which has promoted the exploration of FTO as a potential therapeutic target over the past decade. To date, several FTO inhibitors have been reported, and their therapeutic potency has been evaluated in various cancers types (Fig. 5), as summarized in Table 3.

**Table 3:** The reported inhibitors of FTO listed according to their identified date.

Inhibitor	Year	IC_50_ (μM)	Detection method	Mode of action	Target	Verified substrates	Verified system	Cancer	Ref.
Rhein	2012	21	FP assay	m6A-substrates binder	FTO	m6A ssDNA/RNA	4T1 cell	Breast cancer/AML	^137^^,^^138^^,^^139^
MO-I-500	2014	8.7	Enzyme activity	NA	NA	m6A RNA/microRNA	SUM149 cell	Breast cancer	^140^^,^^141^
MA/MA2	2015	MA:8	Enzyme activity	FTO substrate-site binder	FTO	m6A RNA	A172/U251/U87 cell	Cervical cancer/Glioma	^142^^,^^143^
R-2HG	2018	133.3	Bot blot	2-OG mimetics	FTO	m6A RNA	NOMO/U937 cell	Leukemia/Glioma	^144^^,^^145^
FB23/FB23-2	2019	0.06/2.6	HPLC	FTO substrate-site binder	FTO	m6A/m6Am RNA	NB4/Mice	AML	^146^
Clausine E	2019	27.79	LC/MS	FTO substrate-site binder	FTO	m6A RNA	NA	NA	^147^
Nafamostat mesilate	2019	13.77	LC/MS	FTO substrate-site binder	FTO	NA	NA	NA	^148^
Entacapone	2019	3.5	LC/MS	FTO substrate-site binder	FTO	m6A RNA	Hep-G2 cell/Mice	NA	^149^
CS1/CS2	2020	0.14/0.71	m6A demethylase assay	FTO substrate-site binder	FTO	m6A RNA	NOMO-1/Mice	AML	^150^
Dac51	2021	0.4	HPLC/Dot blot	FTO substrate-site binder	FTO	m6A RNA	B16-OVA/LLC cell/Mice	Melanoma/Lung cancer	^151^
FTO-02/04	2021	2.18/3.39	FEI assay	FTO substrate-site binder	FTO/ALKHB5	m6A/m6Am RNA	TS55576/GBM-GSC-23/GBM-6 cell	Glioblastoma	^152^
18097	2022	0.64	HPLC-MS/MS	FTO substrate-site binder	FTO	m6A	231 cell/Mice	Breast cancer	^153^

2-OG: 2-oxoglutarate; FEI: Fluorescence enzymatic inhibition.

Following the discovery of FTO's demethylase activity, Chen et al identified the first FTO inhibitor, Rhein, through structure-guided and biochemical evaluation. This compound competitively binds to FTO, thereby preventing the recognition of m6A modified RNA and inhibiting the function of FTO.^137^ Furthermore, Yan et al found that the m6A levels are down-regulated in tyrosine kinase inhibitor (TKI) resistant cells. Rhein was shown to increase m6A levels and reduce cell proliferation in NilotinibR and PKC412R-treated leukemia cells.^138^ A recent study demonstrated that Rhein exerts an antitumor effect when integrated with azetidine in AML. Mechanistically, Rhein targets FTO to suppress the AKT/mTOR pathway, thereby reducing multidrug resistance in AML.^27^

FTO catalyzes the m6A demethylation in a Fe^2+^ and 2-oxoglutarate (α-KG) dependent manner. Therefore, modulating the levels and binding of Fe^2+^ and α-KG to FTO represents a significant strategy for inhibiting the function of FTO. The mimic ascorbic acid (MO-I-500) has been reported to inhibit FTO function via suppressing α-KG-dependent hydroxylases.^141^ Singh et al demonstrated that MO-I-500 inhibits cell survival and colony formation via mediating FTO levels.^140^ However, the above inhibitors also suppresses the activity of all α-KG oxygenases. Another FTO inhibitor, R-2HG, which is structurally similar to α-KG, inhibits a broad spectrum of Fe^2+^/α-KG-dependent dioxygenases. They revealed that R-2HG mediates MYC/CEBPA expression in an R-2HG/FTO/m6A axis and plays an anti-proliferation effect in both leukemia and glioma.^144^

In addition to the aforementioned methods, small-molecular compounds that directly bind to FTO also effectively suppress its function. In 2015, Huang et al identified the first FTO special inhibitor meclofenaminc acid (MA), which competes with m6A-modified substrate to bind with FTO. MA2, the ethyl ester derivative of MA, exhibits improved cell penetration and notably decreases the m6A levels.^142^ Xiao et al demonstrated that MA2 enhanced the effect of temozolomide in suppressing glioma cell proliferation by modulating the expression level of FTO downstream target MYC.^143^ Furthermore, numerous studies have confirmed that structure-guided design is an effective strategy for the development of inhibitors.^154^ In 2019, several inhibitors were reported, including a clinical drug, an alkaloid, and optimized MA-derived- compounds. Among them, entacapone was identified as a chemical suppressant of FTO, selected from a pool of 1323 US. Food and Drug Administration-approved drugs. The study revealed that entacapone mediates gluconeogenesis and thermogenesis in an FTO-FOXOA regulatory axis.^149^ Wang et al combined thermodynamic and enzymatic activity methods to reveal the binding of Clausine E with FTO.^147^ Furthermore, Huang et al introduced a 5-membered heterocyclic ring into MA to develop new FTO inhibitors. Among these, FB23 exhibits significantly stronger inhibition on FTO demethylation than MA. Its derivative, FB23-2, significantly inhibits proliferation and promotes the differentiation of AML cells *in vitro,* while also suppressing leukemogenesis in AML mouse models.^146^ Xu et al also confirm the role of FB23-2 in inhibiting tumorigenesis and extending survival in patient-derived xenotransplantation model mice via targeting FTO.^155^ In 2020, Su et al screened 260,000 compounds in the NCI DTP library using a structure-based method, and discovered CS1/CS2, which bind to FTO in the catalytic pocket, inhibiting its demethylase activity. Compared to MO-I500 and FB23-2, CS1 and CS2 demonstrate greater efficacy in suppressing AML cell growth, along with lower IC_50_ values.^150^ Chen et al found that CS2 inhibits oncogenic role of FTO in HCC and enhances the infiltration of macrophages.^51^ FTO drives immune escape by mediating glycolytic metabolism in CD8^+^ T immune cells. To develop an effective FTO inhibitor for the tumor microenvironment, Liu et al optimized FB23 and FB23-2 to develop a new FTO inhibitor, Dac51, which dampens glycolysis in tumor cells and increases the T cell-mediated antitumor effects in an FTO-dependent manner.^151^ To identify clinically applicable inhibitors, Huff et al synthesize a pyrimidine-based FTO inhibitor FTO-02/FTO-04 in a combination of molecular docking and structure-based design methods, which improved m6A and m6Am levels in glioblastoma stem cells (GSCs). They revealed that FTO-04 reduced self-renewal in GSC-derived neurospheres.^152^ In recent years, research has progressed beyond merely identifying existing compounds for FTO inhibition, focusing on the modification of these compounds to enhance their inhibitory effects. Xie et al screened 200,000 compounds using AutoMD, identifying two potential FTO inhibitors, AE-462 and AE-652, which bind to the active domain of FTO to reduce its demethylase activity. They subsequently optimized the structures of AE-462 and AE-652, obtaining the FTO inhibitors 18077 and 18097. Moreover, they demonstrated that 18097 inhibits breast cancer progression both *in vitro* and *in vivo*.^153^ Although existing research demonstrates that targeting the active site of enzymes with small-molecule compounds is a valid manner to modulating the function of oncogenes in cancer, further studies are necessary to realize the application of FTO inhibitors in cancer therapy.

Among the FTO inhibitors identified to date, their application in cancer therapy remains predominantly limited to breast cancer at present. Fernand et al found that Rhein, a FTO inhibitor, acts as an antitumor regulator in breast cancer cells (MCF-7 and MDA-MB-435) through suppressing the activation of VEGF-dependent PI3K, p-AKT, and p-ERK *in vitro.* This suppression results in the inhibition of cell migration, invasion, cell cycle arrest, tumor formation, and the induction of apoptosis.^156^ The anti-proliferative and pro-apoptotic role of Rhein also been demonstrated in HER2 cells through enhancing caspase-9-mediated apoptosis and activating NF-κB and p53-signaling pathways.^139^ The *in vivo* study indicated that Rhein enhances the anti-proliferative effects of atezolizumab in 4T1 cells, leading to a reduction in cancer xenograft growth in mice.^157^ Additionally, meclofenamic acid has been shown to exhibit antiproliferative activity in MCF-7 cells.^158^ Further clinical trials are needed to drive the application of FTO inhibitors in GICs therapy. Combination therapy may represent the most expedient approach to investigate this purpose.

## Conclusions and prospects

Increasing evidence demonstrates that abnormal expression of FTO is significantly associated with cancer occurrence and development. FTO functions not only as an oncogenic factor but also acts to inhibit tumor progression in GICs. Five major cancer-promoting pathways have been reported: (i) FTO demethylates the m6A modification of oncogenes such as *MYC* and *DDIT3,*^48^, ^49^, ^50^ which enhances their mRNA stability and translation efficiency, thereby promoting the proliferation of cancer cells; (ii) FTO modulates metabolic reprogramming by regulating the m6A levels of metabolism-related genes (*GLUT1*, *PKM2*,^52^ and *HK2*^63^), thereby promoting cancer progression; (iii) FTO inhibits the activity of T cell by mediating the m6A modification of the immune checkpoint factor *PD-L1*, contributing to cancer immune escape.^59^ (vi) FTO mediates drug resistance through reducing the m6A levels of chemoresistance-related gene *NEDD4*.^68^ (v) FTO counteracts ferroptosis in CRC cells, promoting tumorigenesis via enhancing the expression levels of solute carrier family 7 member 11 (SLC7A11) and glutathione peroxidase 4 (GPX4).^58^^,^^159^ Conversely, FTO can improve cancer cell stemness,^89^ facilitates metabolic reprogramming,^63^^,^^160^ reduce m6A modification of antioncogenes,^64^ and enhances ferroptosis^159^ to suppress the development of GICs. The current understanding of regulatory networks is insufficient to distinguish the bidirectional role of FTO in GICs. The application of artificial intelligence may offer new insights^41^ to establish a clear classification method.

In addition to its role as an m6A demethylase that influences various biological processes, such as viral infection,^161^ DNA damage,^162^^,^^163^ and stress response, via modifying m6A levels,^164^, ^165^, ^166^ FTO also mediates cellular metabolism in an m6A(m)-independent manner. Kim et al found that FTO mediates canonical WNT/β-Catenin signaling via downregulating DKK1 in an RNA methylation-independent manner, thereby promoting cell migration.^167^ These findings highlight the necessity of exploring multiple perspectives in the function and regulatory mechanisms of FTO in future research.

Despite significant advances in the development of FTO regulators, various aspects warrant further exploration. (i) As a primary m6A demethylase, FTO not only function as an oncogene in GICs, but also has been shown to inhibit cancer progression across various malignancies. To date, nearly all reported FTO regulators are designed to suppress its pro-tumor function based on its substrate and structural characteristics. For instance, Wang et al found that NADP directly binds to FTO, enhancing its activity.^168^ Additionally, Huang et al demonstrated that regulating NADPH levels with branched-chain amino acids (BCAA) could mediate FTO expression.^169^ However, no related FTO activators have been identified so far. Therefore, further studies are needed to elucidate the structural basis and regulatory mechanisms of FTO's function to develop dual-action regulators. (ii) As an m6A and m6Am demethylase, the sensitivity and specificity of FTO regulators warrant closer attention. (iii) Previous studies have demonstrated that FTO inhibitors, such as MO-I-500, CS1/CS2, and 18097, can effectively inhibit FTO activity in breast cancer. Dac51 has been shown to synergize with anti-PD-L1, promoting T cell infiltration.^151^ However, further clinical trials are still needed to validate their clinical practicability. (vi) With ongoing research, the regulatory mechanisms of FTO are being progressively elucidated, encompassing upstream regulators and PTMs, which provide new insight for the developing of FTO regulators to accelerate the advancement of relative drug development.

In summary, FTO plays a crucial role in GICs, which may serve as a potential diagnostic biomarker and therapeutic target. We anticipate that future studies will further reveal its regulatory mechanisms, facilitating the development of both inhibitors and activators, and ultimately advancing their application in clinical treatment.

## CRediT authorship contribution statement

**Xinxin Zhang:** Writing – original draft, Resources, Project administration. **Yufeng Han:** Writing – review & editing, Investigation. **Huimin Yin:** Writing – review & editing. **Zhenjian Zhuo:** Writing – review & editing. **Jing He:** Writing – review & editing.

## Funding

This study was supported by grants from the 10.13039/501100001809National Natural Science Foundation of China (No. 32300473, 82002636, 82173593), 10.13039/501100021171Guangdong Basic and Applied Basic Research Foundation (China) (No. 2023A1515220053), and the Science, Technology and Innovation Commission of Shenzhen, China (No. JCYJ20220531093213030).

## Conflict of interests

All the authors declare that they have no competing interests.
